# Novel *VCP* gene mutation in three siblings with frontotemporal dementia and amyotrophic lateral sclerosis in a Turkish family

**DOI:** 10.1002/alz.086233

**Published:** 2025-01-03

**Authors:** Murat Gultekin, Ayse NazlI Basak

**Affiliations:** ^1^ Erciyes University School Of Medicine, Department of Neurology, Kayseri Turkey; ^2^ Koc University, Department Biology and Genetics, Istanbul Turkey

## Abstract

**Background:**

Valosin Containing Protein (VCP) mutations are responsible some genetic etiologies of amyotrophic lateral sclerosis (ALS) and frontotemporal dementia (FTD).

**Method:**

A 67‐year‐old, male patient, applied to the clinic due to behavioral changes and difficulty swallowing. According the patient history it was reported that his first complaint started 6 years ago (at the age of 61). The patient’s irritability, behavioral and character changes increased over time. The patient consulted a psychiatrist and diagnosed depression then, antidepressant treatment was started.

Genetic investigation was performed for the preliminary diagnosis of FTD‐ALS association. First, the C9orf72 gene report was normal. Then, all exon sequencing was performed. A heterozygous missense mutation p.Arg453Trp (c.1357C>T) was detected in the 11th exome of the VCP gene. This mutation, which was identified for the first time, was also detected in the other two sisters of the patient who had similar complaints.

**Result:**

The patient’s premorbid personality traits were conservative, helpful, social and interested in his family. However, it has been stated that in recent years his sexual desire has increased and he has been making inappropriate conversations in social environments. Difficulty in swallowing was added to the complaints within a year, and in the last two years, speech disorder such as slurring words, increase in appetite, desire to travel and spending money were added.

There was no known history of neuropsychiatric disease in his parents. Three of the four siblings had a similar clinical picture. On examination, the patient’s speech was dysphonic, fasciculation and atrophy was seen in the tongue. 4/5 motor muscle strength in the neck muscles were detected. There was bilateral Hoffman positivity and deep tendon reflex brisk in the lower extremities. Swallowing reflexes could not be obtained. Brain magnetic resonance imaging scan revealed atrophy in the frontotemporal regions. ENMG examination was compatible with motor neuron disease.

**Conclusion:**

ALS and FTD have been considered for many years as two different pathologies with distinct pathology. But, in recent years some studies have revealed that these disorders belong to a continuum, the so‐called ALS‐FTD spectrum disorders. Many causative genes are shared in both diseases.

